# Immunotoxicity and Anti-Inflammatory Characterizations of Prenylated Flavonoids—The Lipophilic 7-*O*-Terpenylated Wogonin

**DOI:** 10.3390/life12122116

**Published:** 2022-12-15

**Authors:** Jin-Yi Wu, Lih-Geeng Chen, Chia-Wen Hu, Kuan-Chi Chiu, Wenhsin Lin, Pei-Chun Ho, Brian Bor-Chun Weng

**Affiliations:** 1 Department of Microbiology, Immunology, and Biopharmaceuticals, College of Life Sciences, Chiayi University, Chiayi 60000, Taiwan; 2 Administration Center of Research and Education, Ditmanson Medical Foundation Chiayi Christian Hospital, Chiayi 60002, Taiwan; 3 iReal Biotechnology, Inc., Hsinchu 300003, Taiwan

**Keywords:** wogonin, survivin, *iNOS*, nitric oxide, terpene, terpenylated flavonoids, RAW264.7 macrophages, lymphocyte, inflammation, cell cycle, Huang-Qin (*Scutellaria baicalensis* Georgi), immunity

## Abstract

Wogonin, one of the exceptional bioactive flavonoids found abundant in the roots of Huang-Qin (*Scutellaria baicalensis* Georgi), is a popular health-preserving Chinese medicine. The therapeutic applications can be expanded by improving its bioavailability. The 7-*O*-terpenylated wogonin consisting one to three prenyl units are chemically synthesized for increasing lipophilic nature for efficient uptake, and also an attempt in mimicry of naturally scarce terpenylated flavonoids found in limited plant families and bee propolis. Wogonin (W) and its lipophilic nature prenyl wogonin (W5), geranyl wogonin (W10), and farnesyl wogonin (W15) were comparatively studied with structure-relationship in immunotoxicity of cell livability on lymphoid, myeloid, and somatic origins cell lines. Anti-inflammatory functions characterized with nitric oxide inhibition and intracellular ROS level of LPS-activated murine macrophage RAW264.7 were assessed. Wogonin and its terpenylated derivatives have selectively influenced livability of lymphoid origin cells but not myeloid and somatic origin cells. The mitotic protein survivin gene expressions analysis further supported the selective suppressions on lymphoid origin YAC-1 cells by wogonin and geranyl wogonin, while oppositely boosted *survivin* expressions in LPS-activated macrophages. Moreover, wogonin exhibits dose-dependent inhibition on the nitric oxide (NO) production and *iNOS* gene expressions of LPS-activated RAW264.7 cells. Terpenylated wogonin exhibits profoundly superior control in intracellular ROS level and a sustained action with sound cell integrity than the wogonin. The enhanced cellular uptake with higher lipophilicity to membrane of 7-*O*-terpenylated wogonin may pose an important biological nature in facilitating better bioavailability and specific immunomodulatory actions of the category of terpenylated flavonoids. The 7-*O*-terpenylated wogonin having biological merit of fast membrane lipid bilayer integration, lower effective concentration, and better preserving immune cells functions and livability deserved further in-depth investigations and their broadly therapeutic applications.

## 1. Introduction

Huang-Qin (*Scutellaria baicalensis* Georgi) is harvested for its fresh root with annual yield about 17 tons in Taiwan. The dry roots slice of Huang-Qin belongs to nutraceutical can be easily accessed from supermarkets is a popular use botanical herb, commonly prepared with four-entity {sì wù} chicken soup as a summer health-preserving formula in Chinese society. Recently, Huang-Qin is renowned as a critical ingredient in the Chinese herbal compound medicine NRICM101 (清冠一號) in treating COVID-19. The baicalein, baicalin and wogonin are the three major flavonoids identified in Huang-Qin that only wogonin exhibit potent anti-inflammatory property by inhibiting both gene expressions of *iNOS* (induced nitric oxide synthase) and *COX-2* (cyclooxygenase-2) in LPS-activated RAW264.7 macrophages [1]. Particularly, the COX-2 gene inhibition property has also been demonstrated in human lung epithelial cancer cells [2], and wogonin has also shown to prevent LPS-induced lung inflammation via activation of PPARγ (peroxisome proliferation-activated receptor γ) [3]. Nevertheless, wogonin also possess many other potent therapeutic functions in anti-cancer, anti-viral infection, and neuroprotection etc [4,5] in additional to its anti-inflammatory property.

Wogonin is currently under intensive studies for its anti-cancer activity primarily evidenced by induction of oxidative stress in attribution to cell apoptosis. Acting as an inhibitor of apoptosis, survivin is not only a normal cell cycle protein expressed in healthy mitotic cells, but also notoriously as a cancer marker highly expressed in progressive cancers [6]. It has been ubiquitously reported wogonin is able to attenuate survivin activity in various cancer cells models [7,8,9]. Nevertheless, survivin plays important role in immune system. It is essential for proliferation and differentiation of normal lymphocytes and macrophages. Lymphoblastogenesis is a fundamental effector response of antigen-activated lymphocytes in mediating acquired immunity. It has been reported survivin knocked out has completely blocked B cell development arrested at pre-B cell stage and impaired B cell clone expansion [10]. Moreover, T cell-specific surviving-deficient mice have mainly immature T cells and diminished peripheral T cells population indicates an important role in T cell development [11]. Survivin has also been reported to express in LPS or IL-4-activated macrophages with M2 polarization [12]. Whether cytotoxic and apoptotic roles of wogonin in cancer cells could be deleterious to mitotic lymphoblastogenesis as fast proliferating lymphocytes in responding to antigen stimulation have to be monitored for potential immunotoxicity.

Although wogonin exhibit multiple therapeutic functions, its bioavailability is low as demonstrated in pharmacological study. The deglycosylated wogonin is not easily absorbed due to low solubility in water, oral bioavailability is only 1.1% [13]. On the other hand, wogonosides of glycosylated form wogonin can quickly mount a high concentration within hours in rat post oral administration [14]. To improve its proficiency, various hydrophilic carriers are available such as polyethylene glycol to improve the water insoluble compound such as wogonin. Hence, to improve wogonin bioavailability and optimizing delivery are under intensive studies and reported [15,16]. Using LC-MS/MS tracking wogonin absorption in beagle dogs shows limited blood uptake and can be improved by hydrophilic carrier arginine solution [15]. Previously, chemically synthesized wogonin derivatives at the 7-*O*-position of the A ring with different number of prenyl units exhibiting enhanced anti-cancer activity by arrest S phase via an apoptotic mechanism [17]. The lipophilic property of the prenyl conjugation to wogonin has been claimed to contribute to the enhanced anti-cancer function. The outcomes in anti-cancer with promoting wogonin bioavailability by increasing lipophilic activity with flavonoid terpenylation has shown some promised results.

Chemically synthesized terpenylated wogonin derivatives has posed some new indications in its bioavailability and delivery. In addition, the terpenylation can also expand biological exploration of the category of natural scarce terpenylated flavonoids. Naturally, majority of flavonoids is *C*-terpenylated, the *O*-terpenylation is even rare and most of them occur as secondary metabolites synthesized in response to pathogenic microbial infection or challenge of abiotic elicitors in some plants [18]. Regardless the structural diversified terpenylated flavonoids have been only found in limited plant families of Leguminosae, Moraceae, Asteraceae of mainly anatomical sections of roots and barks [18], terpenylated flavonoids are also identified in anti-microbial sealant, propolis, of bee hive [19,20]. To learn from nature, chemically synthesized terpenylated flavonoids are of our interest in the perspective of a mimicry to nature and together to promote bioavailability of the anti-inflammatory nature of wogonin for better therapeutic applications. The chemically synthesized 7-*O*-terpenylated wogonin are therefore deemed for further investigations. Currently, the immunotoxicity and anti-inflammatory characteristics are assessed in facilitating scientific basis for its future development in immunomodulatory purposes.

## 2. Materials and Methods

### 2.1. Purification of Wogonin and Synthesis of Terpenylated Wogonin Derivatives

**Scheme 1:**
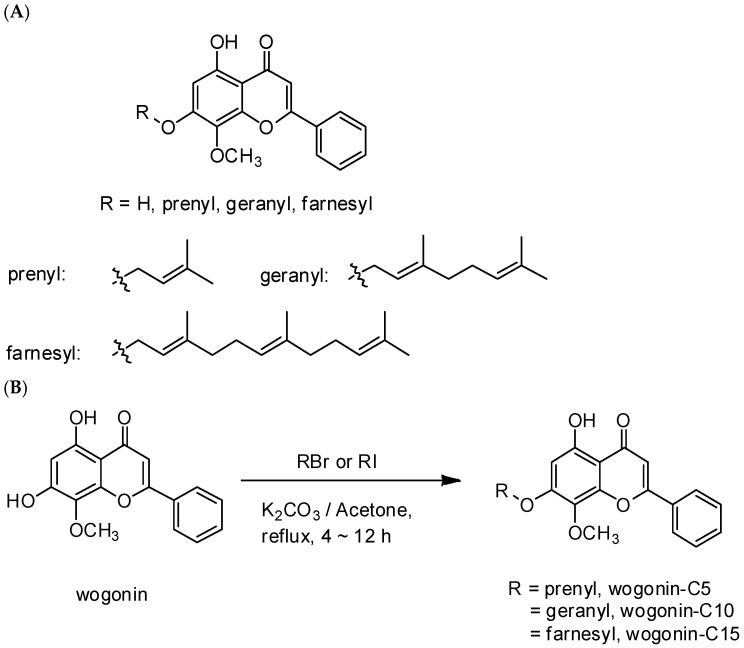
Chemical structures of wogonin and its derivatives (**A**) and synthesis of 7-*O*-terpneyl derivatives of wogonin (**B**).

Wogonin (W) was extracted from dried *Scutellaria baicalensis* Georgi roots that were cut into small pieces, immersed, and extracted with 10-times *v*/*w* acetone twice at room temperature for 2 weeks. After filtration, the residues were reflux-extracted with 4-times *v*/*w* 50% aqueous ethanol twice. Acetone and 50% aqueous ethanol extracts were concentrated, then these extracts were subjected to column chromatography on silica gel eluted with CHCl_3_ and CHCl_3_-MeOH and rechromatographed on silica gel eluted with hexane-acetone to yield W. The readily available W was the starting material for the preparation of terpenylated wogonin derivatives with different units of isoprene. Treatment of W with prenyl bromide, geranyl bromide, and farnesyl bromide in the presence of K_2_CO_3_ gave hemiterpene (prenyl wogonin; W5), monoterpene (geranyl wogonin; W10), and sesquiterpene (farnesyl wogonin; W15) wogonin derivatives, respectively. A scheme for the chemical synthesizing 7-*O*-prenyl derivatives of W is demonstrated in Scheme 1.

### 2.2. Antioxidative Capacity

#### 2.2.1. DPPH Assay

The quenching of free radicals by wogonin and wogonin derivatives evaluated spectrophotometrically at 517 nm against the absorbance of the DPPH (1,1-diphenyl-2-picrylhydrazyl) radical. A freshly prepared DPPH solution (10 mM) was used for the assay. Samples were dissolved in ethanol and the ethanolic solution of DPPH served as a control. The degree of decoloration indicates the free radical scavenging efficiency of the substances. Quercetin and trolox were used as a reference free radical scavenger.

#### 2.2.2. TEAC Assay

ABTS (2,2′-azino-bis-(3-ethylbenthiazoline sulfonic acid)) was used in conjunction with peroxidase and hydroperoxide to generate an oxidized form (ABTS radical). The aliquot of W or respective synthesized derivatives were evaluated against the ABTS radical substrate. The inhibition on the blue greenish color formation was detected with UV-vis absorbance wave length = 734 nm. A trolox standard curve was plotted to obtained relative antioxidative capacity.

### 2.3. Immunotoxicity Assessment

#### 2.3.1. Cell Culture

Murine cell lines of myeloid origin RAW264.7 macrophages (BCRC#60001) and lymphoid origin YAC-1 cells line (BCRC#60147l) and somatic cell line, BNL CL2 embryonic liver cell line (BCRC#60180) were purchased from Bioresource Center and Research Center of the Food and Industrial Research Institute, Taiwan. The cell line was maintained and sub-cultured as recommend. Sub-cultured cells used for the entire study were not exceeding 10th passages. Fresh culture medium was substituted together with transferring into 24-well or 96-well culture plates one day before assay. The seeding density was 1 × 10^6^/well or 2 × 10^5^/well, respectively, for cytotoxic assay and cell cycle analysis. Culture medium was DMEM (Dulbecco’s modified Eagle’s medium) or RPMI 1640 (Roswell Park Memorial Institute) basal medium, which was containing 2 mM L-glutamine and 10% fetal bovine serum (Sigma, Co., Ltd, St. Louis, MO, USA) for the anchorage-dependent or -independent cells uses.

#### 2.3.2. Cell Viability Assay and Microscopic Examination

Cell viability was assessed by staining with 3-(4,5-dimethylthiazol-2-yl)-2,5-diphenyl tetrazolium bromide (MTT), the murine macrophages cell line, RAW 264.7 cell, was plated at a density of 1 × 10^5^ cell/well into 96-well plates. After an overnight conditioning, cells were treated with different concentrations of terpenylated wogonin derivatives for 24 h. At the end of treatment, 20 μL MTT (5 mg/mL) was added, and cells were incubated for 3 h prior to the colorimetrical determination by ELISA plate reader at a 570 nm wavelength (Bio-Tek Instruments, Inc., Winooski, VT, USA). The results were obtained from three independent tests. Seeding in same density, macrophages cultured with 50 μM of terpenylated wogonin derivatives treatments for 1.5 h were subjected for microscopic examination. Photographs were taken at 400× magnification with a CCD equipped microscope (Olympus CKX 41, Japan). Rod and micelle formations of wogonin and terpenylated wogonin derivatives treatments were resolved over 3 h and thereafter monitoring micelle until last observation at 24 h cell culture.

### 2.4. Anti-Inflammatory Function Assessment

#### 2.4.1. Nitric Oxide Production with Griess Test

Extracellular NO production was predicted as nitrite concentration in cell suspension by the Griess reaction. RAW264.7 were incubated with wogonin and its terpenylated derivatives at concentration of 50 μM and stimulated with 100 ng/mL LPS (lipopolysaccharides, Sigma Chemical Co., St. Louis, MO, USA) for 24 h at 5%, 37 °C, humidified incubator. After incubation, 50 μL supernatant was withdrawn from each well and transferred into another 96-well plate for quantification of NO by reaction with Griess reagent. To each well, 50 μL 1% sulphanilamide and 50 μL 0.1% naphthylethene diamine hydrochloride were added in order and then incubated at dark for 15 min at room temperature. Absorbance at 550 nm of the reactants was analyzed by a 96-well plate reader (Bio-Tek Instruments, Inc., Winooski, VT, USA). A standard curve was constructed by preparation of a series of sodium nitrite solutions from 0 to 200 μM.

#### 2.4.2. Intracellular Oxidative Status of Macrophage

The protection of terpenylated wogonin derivatives on LPS-induced reactive oxygen intermediates productions was measured by DCFH (2′,7′-dichlorofluorescin) oxidized fluorescent products retained in RAW264.7 cells. Cells incubated with 50 μM terpenylated wogonin derivatives and 100 ng/mL LPS were loaded with DCFH-DA (dichlorofluorescin diacetate) dye for detecting the amount of intracellular reactive oxygen species. Analysis were performed at 3, 6, 24, and 48 h. Upon a 10 min incubation with DCFH-DA dye loading at dark, room temperature, cells were quickly immersed into ice-cold water incubation prior to flow cytometry analysis (Becton Dickson, Franklin Lakes, NJ, USA). Data analysis was completed by the CellQuest software (Becton Dickson, Franklin Lakes, NJ, USA). The percentage of cells population exerts fluorescence more than the second log values were considered and the geometrical means of fluorescent intensity were recorded.

### 2.5. Flow Cytometry Analysis for Cell Cycle of YAC-1

Cultured lymphoid YAC-1 cells were harvested at an exponential growth phase, and single cell suspensions containing 1 × 10^6^ cells were treated as described previously in cell livability assay at concentrations of 10, 50 and 100 μM for 24 h. At the end of culture, cells were fixed with 70% alcohol. Cell cycle was monitored using propidium iodide (PI) staining of nuclei. The fluorescence of DNA-bound PI in cells was measured with a flow cytometer (FACScan^®^, Becton Dickson, Franklin Lakes, NJ, USA) and the results were analyzed with ModFit software (Becton Dickson, Franklin Lakes, NJ, USA).

### 2.6. mRNA Expression of Survivin and iNOS by Semi-Quantitative RT-PCR Analysis

Briefly, 1 × 10^6^ cells were harvested at designated experimental stages for RNA extractions. Total RNA was extracted using the Trizol reagent following the manufacturer’s instruction. RNA was reverse transcribed using a cDNA synthesis kit (Promega, Madison, WI, USA). Synthesized cDNA was then amplified with Taq polymerase and specific primers for survivin, *iNOS* (inducible nitric oxide synthase) and GAPDH (glyceraldehyde-3-phosphate dehydrogenase) in a thermal cycler. Optimum conditions for RT-PCR were 54 °C for 1 min, 56 °C for 1 min, and 56 °C for 1 min for 30 cycles for survivin, 35 cycles for *iNOS*, and 30 cycles for GAPDH, respectively, and a final extension at 72 °C for 7 min. The primers used were 5′-CCG GTG AAG GAG ACA CCG T-3′ (sense) and 5′-GTG TTC TGA CCA GGC ACG AT-3′ (antisense) for survivin; and 5′-GTC AAC TGC AAG AGA ACG GAG AAC-3′ (sense) and 5′-GAG CTC CTC CAG AGG GTA GGC T-3′ (antisense) for *iNOS*; and 5′-CAG AGC TCC AAT CAA CTG TCG-3′(sense) and 5′-TAA GTT AGA TCC AGC TCC GAC T-3′ (antisense) for GAPDH. A total of 25 μL of final PCR products was analyzed by electrophoresis on 1.2% agarose gel. The bands were visualized with ethidium bromide and each band was measured and analyzed using Gel-Pro Analyzer^®^ software (Media Cybernetics, Inc., Silver Spring, MD, USA).

### 2.7. Statistics

The statistical analysis was performed using Student’s *t* test where significance was specified as * mark at *p*-value less than 0.05. Except the DPPH and ABTS assays were results of duplicated samples in average. Data were presented as mean ± standard deviation. For flow cytometric analysis of cell cycle, the one-way ANOVA was used flowed by treatment comparison by LSD test (SAS software, 1996).

## 3. Results

### 3.1. Antioxidative Capacity of Wogonin and Terpenylated Wogonin Derivatives

Table 1 presents the IC_50_ of free radical scavenging activities of wogonin (W) and terpenylated wogonin with one (W5), two (W10) and three (W15) prenyl units as assessed in DPPH radicals scavenging and acceptor of the donating protons in ABTS^+•^ assay with positive controls of quercetin and trolox. In general, the antioxidative activity of W and its terpenylated derivatives is relatively lower in comparison with the flavonoid counterpart, quercetin. Wogonin and its terpenylated derivatives have IC_50_ more than 200 μM in ABTS^+•^ assay, while the W10 and W15 were less potent in DPPH radical scavenging activity with IC_50_ over 15 μM than W5 and W had IC_50_ = 9.6 and 10.5 μM, respectively.

### 3.2. Differential Cell Viability on Cells of Myeloid and Lymphoid Cell Origins

#### 3.2.1. The Mitochondrial Respiration Activity in MTT Assay

**Figure 1:**
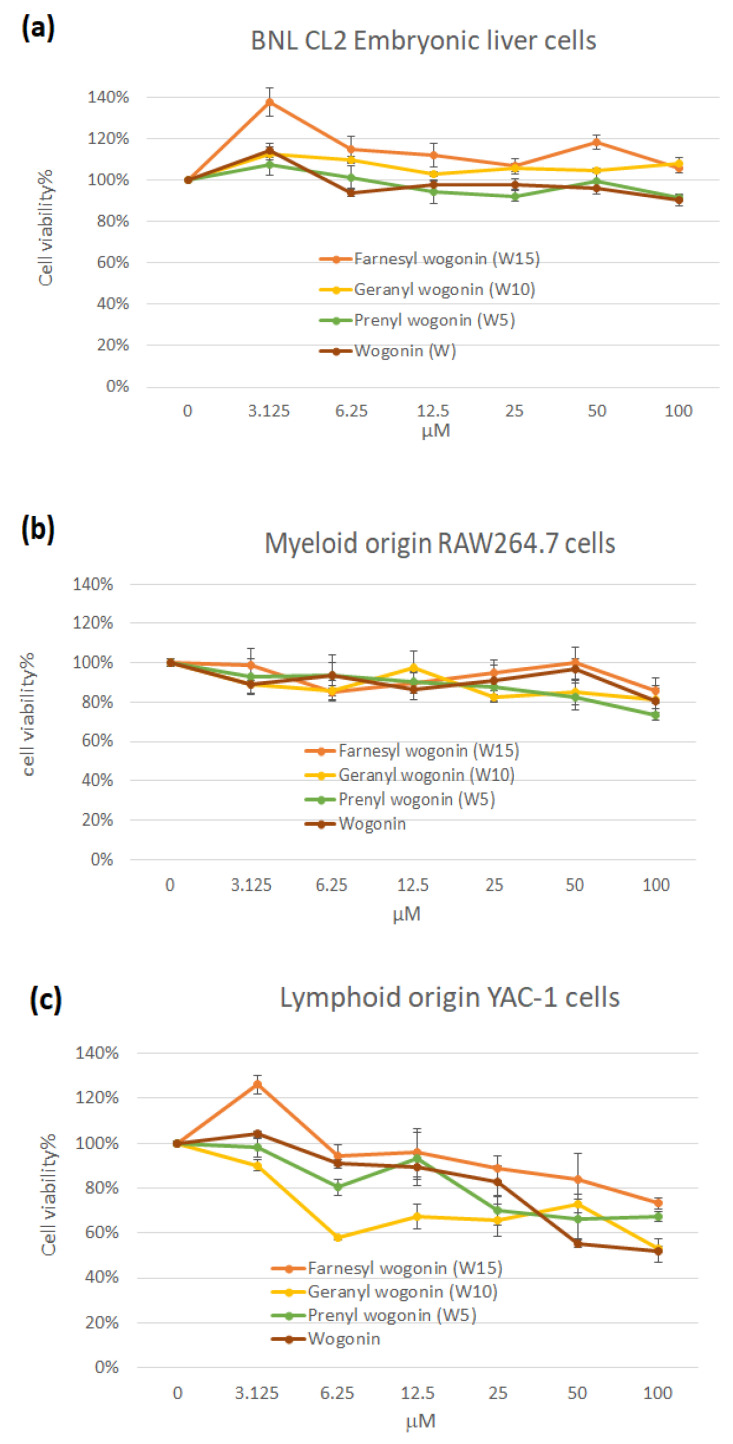
Cell livability of murine somatic cell origin BNL CL2 embryonic liver cells (**a**), the myeloid origin RAW264.7 macrophages (**b**), and lymphoid origin YAC-1 lymphoma cells (**c**) incubated for 24 h with different levels of wogonin (W) or terpenylated wogonin including prenyl wogonin (W5), geranyl wogonin (W10), and farnesyl wogonin (W15).

Three murine cell lines include myeloid origin (RAW264.7), lymphoid origin (YAC-1) and embryonic liver cell (BNL CL2) were either not treated or added with W, W5, W10, or W15 of a serial half dilutions of concentration levels from 100 to 3.125 μM for 24 h co-culture. As assessed by MTT assay, there was no sign of toxicity of wogonin, and its terpenylated derivatives at the maximum level of 100 μM in BNL CL2 liver cells (Figure 1a). Similarly, RAW264.7 macrophages had general cell viability above 80% of all treatments except slightly more reduction to 74% of W5 at 100 μM (Figure 1b). Cytotoxicity to lymphoid origin YAC-1 cells was more obvious of W10 having a faster depression on cell viability at low concentration and leveled out around 53 to 73% when concentration was in a range from 3.125 to 100 μM. All the other treatments exhibited a dose-dependent manner in reducing cell viability but not less than 50% (Figure 1c). At lower concentrations, W10 exhibited higher impact on lymphoid cell YAC-1 viability. In general, cell viability was BNL CL2 > RAW 264.7 > YAC-1 when treated with wogonin or terpenylated wogonin derivatives at concentrations up to 100 μM. Nevertheless, wogonin and its terpenylated derivatives particularly W10 exhibit more influences on lymphoid origin cells than the myeloid or somatic origin cells at the similar concentrations.

#### 3.2.2. Microscopic Examination

**Figure 2:**
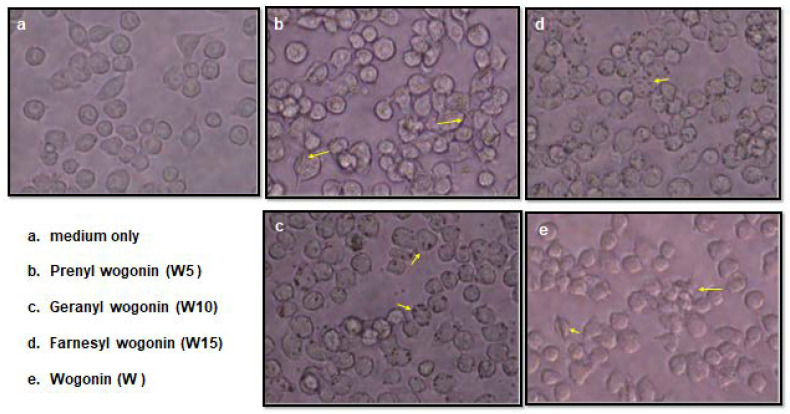
Microscopic examination on the cell morphology of macrophages treated with wogonin and its terpenylated derivatives for 90 min. The spear rod and micelle-like formations accumulated onto the cell′s outer surface are indicated with yellow arrowheads.

The macrophage RAW264.7 treated with wogonin (W), prenyl wogonin (W5), geranyl wogonin (W10), and farnesyl wogonin (W15) at 50 μM for 90 min are shown in Figure 2. As indicated by the yellow arrows, the crystal rod-like objects were seen in wogonin (Figure 2e) and prenyl wogonin (Figure 2b) treated cells, whereas the round micelle-like dark color objects were obviously seen in geranyl (Figure 2c) and farnesyl (Figure 2d) wogonin treatments. All observed entities vanished at the next monitoring time point, 180 min, and thereafter during the extended culture. To explain this phenomenon, the calculated lipophilicity values (clog*P*) value is performed, which is a well-established formula for measuring a compound’s hydrophilicity in analytic chemistry. The longer terpenyl moiety predicted a higher lipophilicity value, which may enhance their membrane lipid bilayer permeability. This is in accordance with microscopic observations that wogonin and terpenylated wogonin derivatives showed a high tendency to accumulate on cells’ outer surface rather than precipitate in the vicinity of cells. The numbers of prenyl units of wogonin *O*-prenylation increase linearly along with the insolubility in culture medium, while higher lipophilic chemistry tends to form the rod or micelle-like structures, which exhibit higher affinity to membrane lipid bilayer as seen mainly accumulating onto cells. Indeed, faster integrations into cells are expected since no obvious cell morphology changes, and precipitate objects were not observed afterward at later microscopic examinations.

### 3.3. Anti-Inflammatory Property

#### 3.3.1. Wogonin Suppress LPS-Induced Nitric Oxide (NO) Production

**Figure 3:**
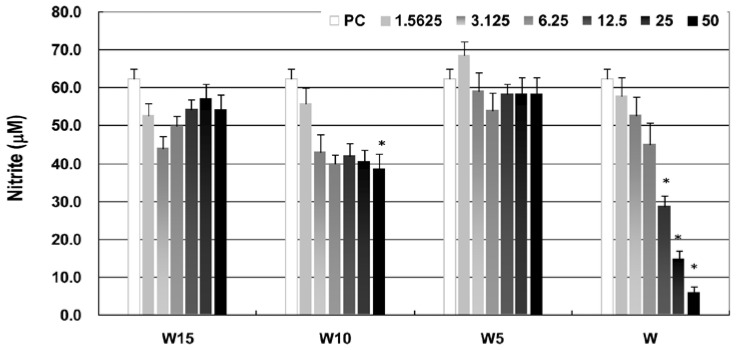
The nitric oxide (NO) production of LPS-activated macrophages treated with 50 μM of wogonin (W), prenyl wogonin (W5), geranyl wogonin (W10), or farnesyl wogonin (W15) for 24 h is shown. The * indicates a significantly different at a *p*-value less than 0.05 in comparison with PC (positive control; medium only of LPS-activated macrophages).

As demonstrated in Figure 3, wogonin (W) exhibits dose-dependently suppressed NO production (*p* < 0.05) in LPS-activated macrophages, while all three terpenylated wogonin derivatives have only moderately inhibited NO production at lower concentrations (<6.25 μM). Particularly, geranyl wogonin (W10) exhibited more effective suppression in a dose-dependent manner at lower doses when compared with the others. Moreover, the NO inhibition plateaued when concentrations were higher than 6.25 μM in geranyl wogonin (W10), whereas the prenyl wogonin (W5) and farnesyl wogonin (W15) gradually lost inhibitory activity at higher concentrations of 6.25 to 50 μM. Nevertheless, there was no sign of NO suppression dependent on the number of isoprene units of terpenylated wogonin. Moreover, the dose-dependent effectiveness of wogonin on suppression of NO production in LPS-activated macrophages is evidenced.

#### 3.3.2. Changes of Intracellular ROS Overtime in LPS-Activated Macrophages

**Figure 4:**
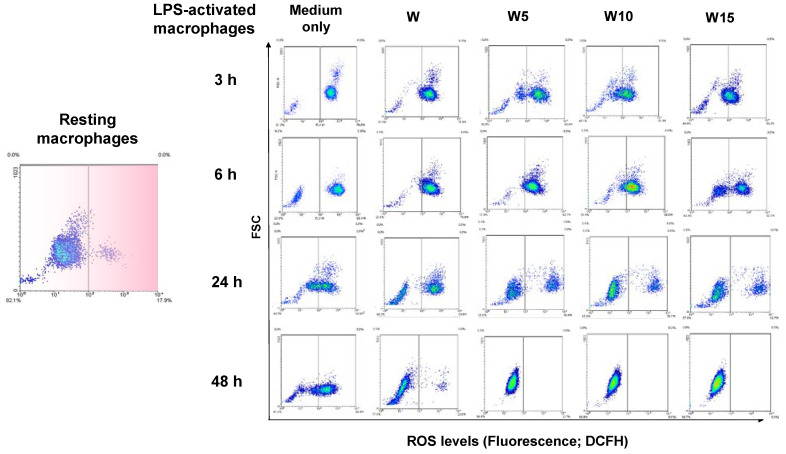
The flow cytometric density plots show the intracellular ROS level of normal resting macrophages and LPS-activated macrophages treated with medium only, wogonin (W), prenyl wogonin (W5), geranyl wogonin (W10), or farnesyl wogonin (W15). The *y*-axis (FSC) represented cell size, and the *x*-axis represented ROS levels of fluorescence generated by oxidized DCF. All treatments were at the same dosage of 50 μM, and LPS was used to agitate macrophages at 100 ng/mL.

The inflamed macrophages are aggressively producing reactive oxygen species, which can be detected intracellularly with flow cytometric analysis on fluorescence changes by DCF (2′,7′-dichlorofluorescin) dye. Continuously monitoring intracellular ROS levels in LPS-activated macrophages treated at a concentration of 50 μM with either W, W5, W10, or W15 are shown in Figure 4. As demonstrated, the resting macrophages have low intracellular ROS levels, and the cell population is mainly aside in the left portion. In the first column of the LPS-activated macrophages cultured with medium only, the cell population shifts to the right with the increasing of ROS levels at 3 h and 6 h and then gradually shift back to the left along with diminishing fluorescence over time. In a similar manner, inflamed macrophages treated with wogonin (W) shows the entire population shifts to the right but lower intracellular ROS levels than the medium-only group at 3 and 6 h. While the majority of cells were returned to the baseline (left side) by 48 h of the W group, LPS-activated, and the medium-only treatment group had the entire cell population still remained in a high ROS level (right side). Interestingly, the suppression of intracellular ROS levels obviously showed isoprene unit numbers-dependent manner over time. The effectiveness is in the order of farnesyl wogonin (W15) > geranyl wogonin (W10) > prenyl wogonin (W5); as seen, the dense cell population is holding back on the left but shifting to the right at 3 and 6 h. Moreover, as seen in all the terpenylated wogonin treatments, two departure populations of distinct ROS levels are noticed by 24 h. Curiously, those inflamed populations even exhibit higher ROS levels than the maximal ROS level observed in the medium-only group. By 48 h, all terpenylated wogonin treatments exhibit a stabilized intracellular ROS level in the entire cell population, while scarce and the majority of inflamed cell populations are still noticeable in W and medium-only treatments, respectively. Moreover, as comparisons in 48 h row, the cell integrity is better retained in terpenylated wogonin-treated macrophages while more cell debris appears in the wogonin and medium-only groups, which might be attributed to the almost immediately obstruct increased ROS production by 3 h and faster calming down elevated ROS by 48 h with terpenylated wogonin treatments.

### 3.4. Cell Cycle Analysis in Lymphoid Origin YAC-1 Cells

**Figure 5:**
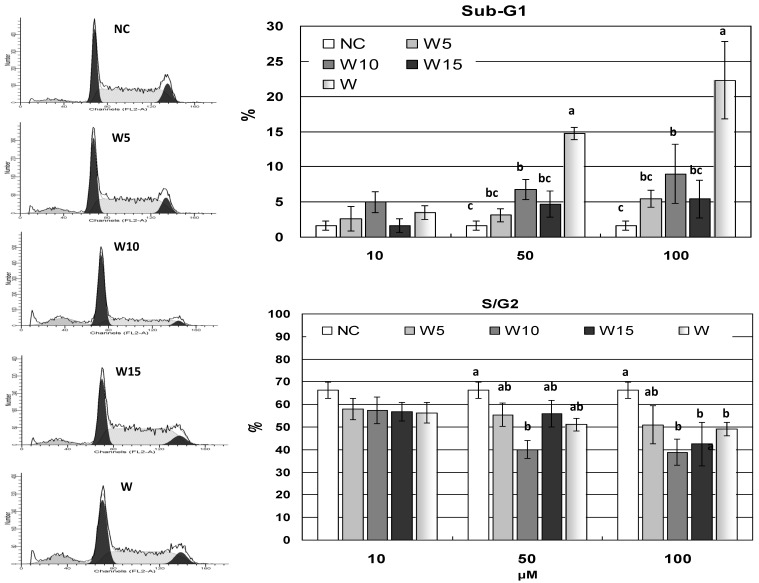
Cell cycle analysis of the lymphoid origin YAC-1 cells incubated with medium only (NC) or different concentrations of wogonin (W), prenyl wogonin (W5), geranyl wogonin (W10), or farnesyl wogonin (W15). Results are expressed as mean ± SD. The different letter between treatments means significance at *p* < 0.05.

The mitotic activity of antigen-activated lymphocytes is critical in facilitating acquired immunity. The impact of anti-inflammatory wogonin and its terpenylated derivatives on the cell cycle of lymphoid origin cell line YAC-1 is assessed, as shown in Figure 5. With the awareness that YAC-1 cells may not best resemble the actual cell behavior of proliferating lymphocyte (lymphoblastogenesis) and is indeed a cancer cell line, the wogonin (W) and geranyl wogonin (W10) treatments have resulted in significant (*p* < 0.05) higher sub-G1 populations than that of the control group (NC) at concentrations of 50 and 100 μM, while the wogonin shows the highest effectiveness in inducing apoptosis and prenyl wogonin (W5) and farnesyl wogonin (W15) show no statistical difference with the control group at all concentrations. Moreover, geranyl wogonin (W10) significantly (*p* < 0.05) depressed the S/G2 phase of the cell cycle in YAC-1 cells at concentrations of 50 and 100 μM as compared with those of the control group (NC), whereas the wogonin (W) and farnesyl wogonin (W15) have exhibited significant (*p* < 0.05) suppression on S/G2 phase at higher concentration 100 μM. On the other hand, the prenyl wogonin shows a moderate impact on the S/G2 phase and no statistical difference in the sub-G1 ratio as compared with that of the control (NC) group.

### 3.5. Gene Expressions Analysis of iNOS and Survivin

**Figure 6:**
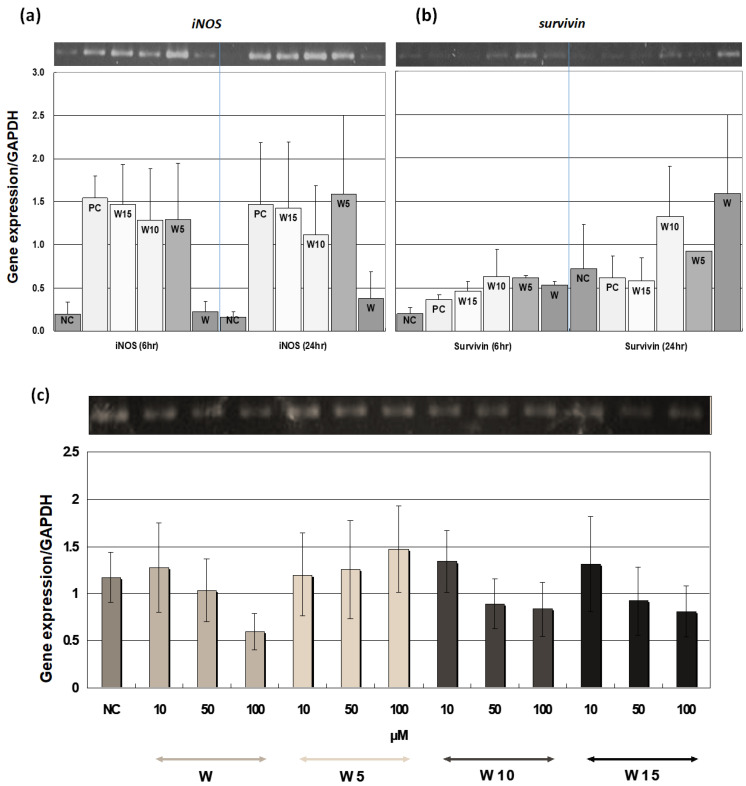
The *iNOS* (**a**) and *survivin* (**b**) gene expressions at 6 and 24 h of macrophage (NC) or LPS-activated macrophages (PC), and treating with 50 μM of wogonin (W), prenyl wogonin (W5), geranyl wogonin (W10), or farnesyl wogonin (W15); *survivin* gene expression of lymphoid origin YAC-1 cells incubated with medium only (NC) or treated different concentrations of wogonin (W), prenyl wogonin (W5), geranyl wogonin (W10), or farnesyl wogonin (W15) (**c**). Data are presented as mean ± SD, *n* = 3.

The gene expressions of *iNOS* and *Survivin* in LPS-activated macrophages are demonstrated with 6 h and 24 h results (Figure 6a). In agreement with previous results on NO production of LPS-activated macrophages, wogonin has effectively inhibited *iNOS* gene expression to comparative levels of negative control (NC) group of 6 and 24 h results. Terpenylated wogonin derivatives show a negligible difference with the positive control (PC), except geranyl wogonin (W10) has averagely lower *iNOS* gene expression than prenyl (W5) and farnesyl (W15) wogonin. On the other hand, *survivin* expressions in LPS-activated macrophages had slightly elevated *survivin* at 6 h but not 24 h of the PC group (LPS only). Expressions of *survivin* were averagely higher in W10, W5, and W groups than those of NC, PC, and W15 groups at 6 h and 24 h, while the increases were more obvious at 24 h. In Figure 6b, the *survivin* gene expressions in lymphoid origin YAC-1 cells at 24 h is shown there was a dose-dependent manner in suppression of wogonin (W), geranyl wogonin (W10), and farnesyl wogonin (W15) at a concentration range from 10 to 100 μM. Moreover, the prenyl wogonin (W5), on the other hand, shows a dose-dependent increase in lymphoid origin YAC-1 cell *survivin* expression. In general, the average survivin gene expressions were similar among all treatment groups when treated at low concentrations.

## 4. Discussion

Terpenylated flavonoids are mainly *C*-prenylated. The *O*-prenylated structures are unique chemicals naturally synthesized in limited plant families of Leuminosae, Moraceae, and Astreaceae, mainly as secondary metabolites in defense against microorganisms [18]. Propolis for building bee hives also exist prenylated flavonoids [20,21], and they may exhibit multiple biological functions, including antioxidant, anti-microbial anti-inflammation, anti-cancer activities, etc. Due to their naturally scarce occurrence, chemically synthesized to promote anti-cancer activity by increasing lipophilic tendency of baicalein, wogonin, and chrysin of 7-*O*-alkylated isoprene have been reported for specific comparisons [17]. Recently, 7-*O*-alkyl or 7-*O*-acyloxy derivatives of natural flavonoids (naringenin and apigenin) have been synthesized chemically to avoid methylation, sulfation, glucuronidation, and phenolation during phase-two metabolism and to enhance their functions [22,23]. Currently, the 7-*O*-alkylated wogonin with one to three isoprene units termed prenyl wogonin (W5), geranyl wogonin (W10), and farnesyl wogonin (W15) have been shown to increase lipophilic activity as observed the preferential membrane-bound rod or micelle-like entities (Figure 2) along with the clog*P* values of the extending terpenyl moieties. A dose-dependently selective immunotoxicity in lymphoid origin YAC-1 cells but not myeloid (macrophage RAW264.7) and somatic (embryonic liver cells BNL CL2) origins are demonstrated (Figure 1). Nevertheless, the overall cell livability of YAC-1 cells remained above 50% at concentrations less than 100 μM of all treatments. The increased lipophilic property of terpenylated wogonin derivatives has not departed from wogonin in cytotoxicity. To further follow these results, cell cycle analysis on YAC-1 cells demonstrates that wogonin at higher concentrations of 50 and 100 μM significantly induced apoptosis by elevating sub-G1 proportion (Figure 5). Wogonin and geranyl wogonin have also reduced the S/G2 proportions, while prenyl wogonin exhibits relatively less impact on the cell cycle, which is in agreement with the finding of sustained mitotic survivin gene expression of W5 (Figure 6c). Wogonin has been shown to preferentially induce malignant lymphocyte apoptosis while no toxicity to normal lymphocytes [24]. It is worth noting that YAC-1 is a cancer cell line that may not well-represent normal lymphoblastogenic lymphocytes. Antigens activate selected lymphocyte clonal proliferation and facilitate fundamental steps in acquired immunity. Hampered lymphocyte proliferation has been shown to disrupt both cell-mediated and humoral immunities. Terpenylated wogonin derivatives exhibit less impact in inducing apoptosis of proliferating lymphoid origin YAC-1 cells might imply additional advantages besides the well-recognized anti-cancer property of wogonin.

Moreover, the anti-inflammatory property as assessed by nitrite concentration of LPS-activated macrophages in wogonin and terpenylated wogonin derivatives shows different degrees of NO (nitric oxide) suppressions (Figure 3), and wogonin exhibits a potent anti-inflammatory property in a dose-dependent manner at concentrations up to 50 μM. At similar concentration levels, Lee and Park [1] have also demonstrated inhibitions of NO and other pro-inflammatory and inflammatory cytokines in polyinosinic-poly-cytidylic acid-induced RAW264.7 macrophages. Compared with wogonin, terpenylated wogonin derivatives show moderate or even no inhibitions on NO productions. Interestingly, at lower concentration levels (1.5625 to 6.25 μM), geranyl wogonin exhibits even faster-depressing NO production than wogonin, and the suppression has leveled at higher concentrations up to 50 μM. Moreover, while geranyl wogonin (W10) has significantly (*p* < 0.05) inhibited NO production at a concentration of 50 μM as compared to the positive control (PC) of medium only of LPS-activated macrophages, prenyl (W5) and farnesyl (W15) wogonin exhibit no inhibitions on NO productions. It is worth mentioning that while W5 and W15 have reduced NO inhibition capacity, they exhibit better maintaining intracellular ROS levels than wogonin in LPS-activated macrophages, as seen in Figure 4. Moreover, coincidently parallel to the effect on reducing cell livability of YAC-1 cells (Figure 1c), the reduction was leveled at higher concentrations of terpenylated wogonin, particularly of geranyl wogonin. Structure-functional activities of flavonoids with isoprenoid chains have been reviewed pharmacologically in favor of increasing affinity to biological membranes [25]. Indeed, flavonoids such as wogonin are chemically amphipathic molecules of both hydrophobic and hydrophilic moieties. Substitution of the hydrophilic hydroxyl (OH) group may reduce the hydrophilic property, alternatively increasing hydrophobic interaction with the cell membrane. The number of isoprene units alkylated to wogonin may add a lipophilic property or micelle-forming property, which may differentially affect the membrane integration or retention in the lipid bilayer of the prenyl, geranyl, or farnesyl wogonin at specific niches. Furthermore, it is important to mention that although they are both biological membranes, cell membrane and nucleus membrane fundamentally evolved differently in the eukaryotes. The former is a single lipid bilayer, and the later nucleus membrane is doubled. As the result of *iNOS* gene expression demonstrated (Figure 6a), it is reasonable to suspect that the different number of isoprene units may serve differently in integration with the two distinct biological membranes. Certainly, the diverse natural occurrences of terpenylated flavonoids with *C*-prenylated or *O*-prenylated conjugates [18] having various combinations of terpenyl length, conjugated position, number of terpenyl conjugates plus the diversified terpenoids, which possess independent biological roles to add more interesting questions await for further elucidations.

Furthermore, the antioxidative activities of wogonin and its terpenylated derivatives as assessed by flow cytometric analysis of intracellular ROS level of LPS-activated macrophages at 3, 6, 24, and 48 h (Figure 4) have revealed the merits of terpenylated wogonin in harnessing elevating ROS over the wogonin. Prenylated flavonoids naturally isolated from Heartwood of *Artocarpus communis* [26] or bee propolis [20] show potent antioxidative and anti-inflammatory activities. Currently, LPS-activated macrophages have almost immediately increased intracellular ROS level since 3 h, maximized at 6 h, and slowly back to resting ROS level after 48 h. As the cell density plot demonstrated, a time-dependent and isoprene unit number-dependent manner on shifting the cell density from right to left is evidenced. As discussed earlier, the higher clog*P* values of the 7-*O*-substituted wogonin derivatives implied higher lipophilicity. By improving membrane affinity, integration, and possible permeability, the structure-function-related intracellular ROS scavenging is attained in terpenylated wogonin. Moreover, the geranyl wogonin (W10) exhibits the best controlling elevating intracellular ROS level of LPS agitated macrophages as shown by a fast recovery to basal ROS level over time when compared with the other treatments. Nevertheless, wogonin exhibits anti-cancer effects by up-regulating intracellular ROS levels to achieve cytotoxicity and apoptosis [17,27,28,29]. The disparity of wogonin in inducing ROS generation in cancer cells or suppression of ROS levels in LPS-activated macrophages implies its complicated role in regulating intracellular ROS levels. The 7-*O*-terpenylated wogonin with different number units of isoprene seems to differentially exhibit functions in the current immune characterizations. Furthermore, although the prenol, geraniol, and farnesol have been tested with no toxicity to current tested cell lines, these terpenes may independently exert potent anti-tumor and anti-inflammation activities [30,31]. Since the intracellular dynamic of terpenylated wogonin has not been tracked, there is no reason to not believe synergistic effects may occur sometime during the extended culture. Although it is still controversial, the terpenes’ antioxidative roles in cells have been reviewed, and isoprene may exhibit multiple regulatory functions in plants other than scavenging free radicals [32]. The current study demonstrated the immunological advantages of chemically synthesized 7-*O*-terpenylated wogonin, particularly geranyl wogonin may act with a lower effective dosage, prolonged action, and better preserves livability and functions of immune cells of lymphoid and myeloid cells origins. In addition, the current data may also support the biological importance of naturally diverse terpenylated flavonoids, which may pose new indications in future drug discovery and applications.

## Figures and Tables

**Table 1:** Radical scavenging activities of wogonin (W) and its 7-*O*-terpenylated derivatives, including prenyl (W5), geranyl (W10), and farnesyl (W15) wogonin, are assessed with ABTS^+•^ and DPPH assays.

Compounds	ABTS^+• 1^	DPPH ^1^
Wogonin (W)	10.5 (1.2)	>200 (<0.03)
Prenyl wogonin (W5)	9.6 (1.3)	>200 (<0.03)
Geranyl wogonin (W10)	>15.0 (<0.1)	>200 (<0.03)
Farnesyl wogonin (W15)	>15.0 (<0.1)	>200 (<0.03)
Quercetin	2.1 (5.5)	7.0 (2.1)
Trolox	12.5 (1.0)	15.0 (1.0)

^1^ Each result is expressed as the IC_50_ value (μM), and the relative value to that of trolox is shown in parentheses.

## Data Availability

The data may be available upon request to the corresponding author.

## References

[1] Lee J.Y., Park W. (2015). Anti-Inflammatory Effect of Wogonin on RAW 264.7 Mouse Macrophages Induced with Polyinosinic-Polycytidylic Acid. Molecules.

[2] Chen L.-G., Hung L.-Y., Tsai K.-W., Pan Y.-S., Tsai Y.-D., Li Y.-Z., Liu Y.-W. (2008). Wogonin, a bioactive flavonoid in herbal tea, inhibits inflammatory cyclooxygenase-2 gene expression in human lung epithelial cancer cells. Mol. Nutr. Food Res..

[3] Yao J., Pan D., Zhao Y., Zhao L., Sun J., Wang Y., You Q.-D., Xi T., Guo Q.-L., Lu N. (2014). Wogonin prevents lipopolysaccharide-induced acute lung injury and inflammation in mice via peroxisome proliferator-activated receptor gamma-mediated attenuation of the nuclear factor-kappaB pathway. Immunology.

[4] Huynh D.L., Ngau T.H., Nguyen N.H., Tran G.-B., Nguyen C.T. (2020). Potential therapeutic and pharmacological effects of Wogonin: An updated review. Mol. Biol. Rep..

[5] Banik K., Khatoon E., Harsha C., Rana V., Parama D., Thakur K.K., Bishayee A., Kunnumakkara A.B. (2022). Wogonin and its analogs for the prevention and treatment of cancer: A systematic review. Phytotherapy Res..

[6] Jaiswal P.K., Goel A., Mittal R.D. (2015). Survivin: A molecular biomarker in cancer. Indian J. Med. Res..

[7] Huang K., Zhang G., Huang Y., Diao Y. (2012). Wogonin induces apoptosis and down-regulates survivin in human breast cancer MCF-7 cells by modulating PI3K–AKT pathway. Int. Immunopharmacol..

[8] Groner B., Weiss A. (2013). Targeting survivin in cancer: Novel drug development approaches. BioDrugs.

[9] Sereshgi M.M.A., Mashayekhi A., Norbazargan H. (2021). Wogonin Stimulation of Cell Death and Reducing Survivin in MDM-MB231 Breast Tumors. Immunoregulation.

[10] Miletic A.V., Jellusova J., Cato M.H., Lee C.R., Baracho G.V., Conway E., Rickert R.C. (2016). Essential Role for Survivin in the Proliferative Expansion of Progenitor and Mature B Cells. J. Immunol..

[11] Xing Z., Conway E., Kang C., Winoto A. (2003). Essential Role of Survivin, an Inhibitor of Apoptosis Protein, in T Cell Development, Maturation, and Homeostasis. J. Exp. Med..

[12] Benaiges E., Ceperuelo-Mallafré V., Madeira A., Bosch R., Núñez-Roa C., Ejarque M., Maymó-Masip E., Huber-Ruano I., Lejeune M., Vendrell J. (2021). Survivin drives tumor-associated macrophage reprogramming: A novel mechanism with potential impact for obesity. Cell. Oncol..

[13] Talbi A., Zhao D., Liu Q., Li J., Fan A., Yang W., Han X., Chen X. (2014). Pharmacokinetics, Tissue Distribution, Excretion and Plasma Protein Binding Studies of Wogonin in Rats. Molecules.

[14] Sharifi-Rad J., Herrera-Bravo J., Salazar L.A., Shaheen S., Ayatollahi S.A., Kobarfard F., Imran M., Imran A., Custódio L., López M.D. (2021). The Therapeutic Potential of Wogonin Observed in Preclinical Studies. Evid.-Based Complement. Altern. Med..

[15] Zhu N., Li J.-C., Zhu J.-X., Wang X., Zhang J. (2016). Characterization and Bioavailability of Wogonin by Different Administration Routes in Beagles. J. Pharmacol. Exp. Ther..

[16] Baek J.-S., Na Y.-G., Cho C.-W. (2018). Sustained Cytotoxicity of Wogonin on Breast Cancer Cells by Encapsulation in Solid Lipid Nanoparticles. Nanomaterials.

[17] Wang S.-H., Chen C.-H., Lo C.-Y., Feng J.-Z., Lin H.-J., Chang P.-Y., Yang L.-L., Chen L.-G., Liu Y.-W., Kuo C.-D. (2015). Synthesis and biological evaluation of novel 7-O-lipophilic substituted baicalein derivatives as potential anticancer agents. MedChemComm.

[18] Barron D., Ibrahim R.K. (1996). Isoprenylated flavonoids—A survey. Phytochemistry.

[19] Huang S., Zhang C.-P., Wang K., Li G.Q., Hu F.-L. (2014). Recent Advances in the Chemical Composition of Propolis. Molecules.

[20] Arung E.T., Pasedan W.F., Tandirogang N., Allam A.E., Amen Y., Shimizu K., Ishikawa H., Syafrizal S., Sukemi S. (2020). Prenylated Flavonoids as Antioxidant and Melanin Inhibitors From Stingless Bee (*Wallacetrigona incisa*) Propolis. Nat. Prod. Commun..

[21] Kumazawa S., Ueda R., Hamasaka T., Fukumoto S., Fujimoto T., Nakayama T. (2007). Antioxidant Prenylated Flavonoids from Propolis Collected in Okinawa, Japan. J. Agric. Food Chem..

[22] Kozłowska J., Grela E., Baczyńska D., Grabowiecka A., Anioł M. (2019). Novel O-alkyl Derivatives of Naringenin and Their Oximes with Antimicrobial and Anticancer Activity. Molecules.

[23] Chu M., Kang D.W. (2020). Synthesis of 7-O-alkyl or 7-O-acyl Derivatives of Naringenin and Apigenin. J. Korean Chem. Soc..

[24] Baumann S., Fas S.C., Giaisi M., Müller W.W., Merling A., Gülow K., Edler L., Krammer P.H., Li-Weber M. (2008). Wogonin preferentially kills malignant lymphocytes and suppresses T-cell tumor growth by inducing PLCγ1- and Ca^2+^-dependent apoptosis. Blood.

[25] Botta B., Vitali A., Menendez P., Misiti D., Monache G. (2005). Prenylated Flavonoids: Pharmacology and Biotechnology. Curr. Med. Chem..

[26] Han A.-R., Kang Y.-J., Windono T., Lee A.S.K., Seo E.-K. (2006). Prenylated Flavonoids from the Heartwood of Artocarpus communis with Inhibitory Activity on Lipopolysaccharide-Induced Nitric Oxide Production. J. Nat. Prod..

[27] Tsai C.-F., Yeh W.-L., Huang S.M., Tan T.-W., Lu D.-Y. (2012). Wogonin Induces Reactive Oxygen Species Production and Cell Apoptosis in Human Glioma Cancer Cells. Int. J. Mol. Sci..

[28] Qian C., Wang Y., Zhong Y., Tang J., Zhang J., Li Z., Wang Q., Hu R. (2014). Wogonin-enhanced reactive oxygen species-induced apoptosis and potentiated cytotoxic effects of chemotherapeutic agents by suppression Nrf2-mediated signaling in HepG2 cells. Free Radic. Res..

[29] Koh H., Sun H.-N., Xing Z., Liu R., Chandimali N., Kwon T., Lee D.-S. (2020). Wogonin Influences Osteosarcoma Stem Cell Stemness Through ROS-dependent Signaling. In Vivo.

[30] Yang W., Chen X., Xiuling Y., Guo S., Wang Z., Yu X. (2020). Advances in Pharmacological Activities of Terpenoids. Nat. Prod. Commun..

[31] Masyita A., Sari R.M., Astuti A.D., Yasir B., Rumata N.R., Emran T.B., Nainu F., Simal-Gandara J. (2022). Terpenes and terpenoids as main bioactive compounds of essential oils, their roles in human health and potential application as natural food preservatives. Food Chem. X.

[32] Pollastri S., Baccelli I., Loreto F. (2021). Isoprene: An Antioxidant Itself or a Molecule with Multiple Regulatory Functions in Plants?. Antioxidants.

